# Supplementary material for: Association between oral microbiome and seven types of cancers in East Asian population: a two-sample Mendelian randomization analysis
Source: Front Mol Biosci. 2023 Nov 21;10:1327893. doi: 10.3389/fmolb.2023.1327893 (PMC10702768; doi:10.3389/fmolb.2023.1327893)
Supplement: Supplementary file 1 [file DataSheet1.ZIP › Supplementary Materials/MR plots for tongue/tongue═╝/Gastric cancer/pheno.101_to_gastric cancer_forest.pdf]

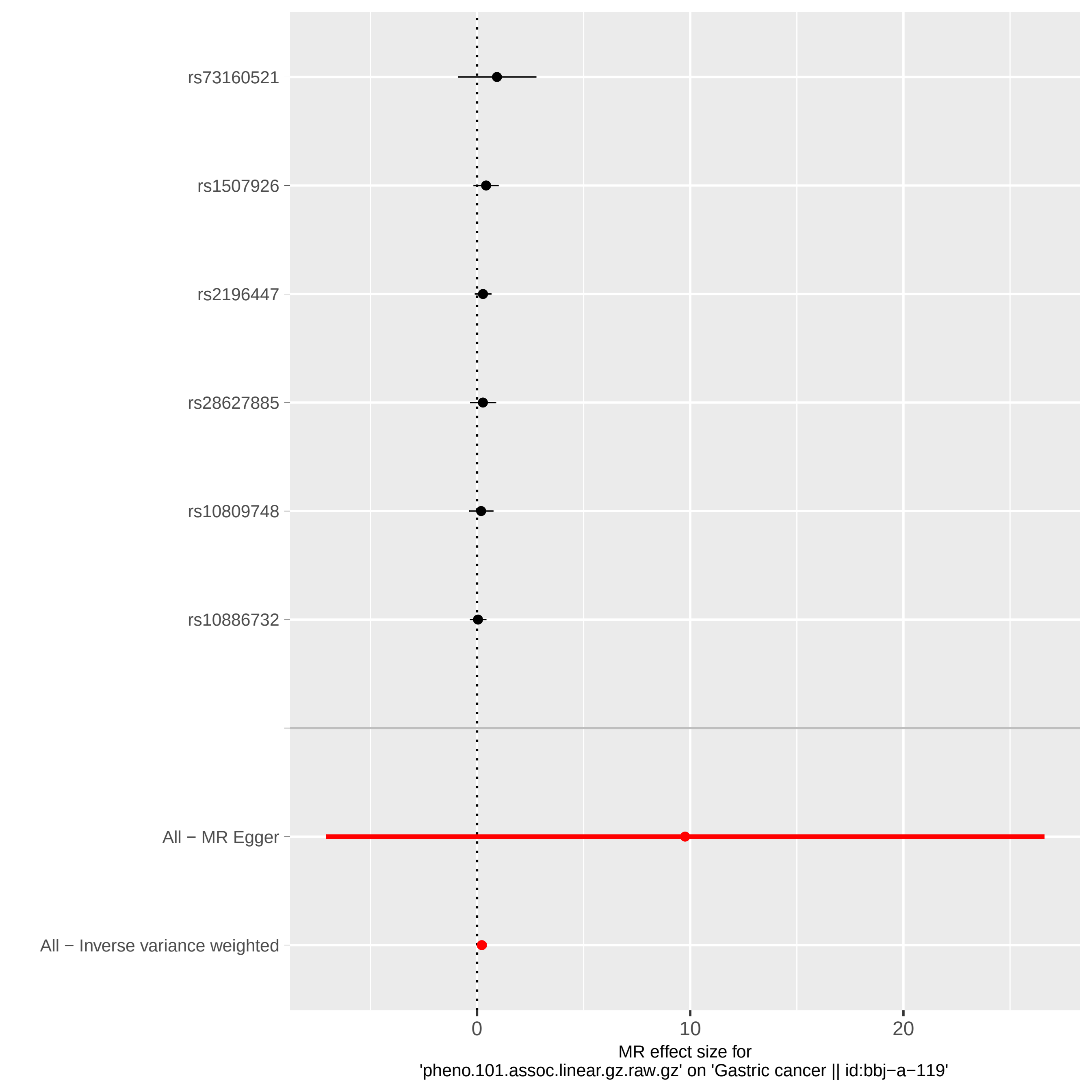
rs73160521
rs1507926
rs2196447
rs28627885
rs10809748
rs10886732
All − MR Egger
All − Inverse variance weighted
0
10
20
MR effect size for
'pheno.101.assoc.linear.gz.raw.gz' on 'Gastric cancer || id:bbj−a−119'
